# Status of health information exchange: a comparison of six countries

**DOI:** 10.7189/jogh.09.020427

**Published:** 2019-12

**Authors:** Thomas H Payne, Christian Lovis, Charles Gutteridge, Claudia Pagliari, Shivam Natarajan, Cui Yong, Lue-Ping Zhao

**Affiliations:** 1 University of Washington, Seattle, Washington, USA; 2 University of Geneva and University hospitals of Geneva, Switzerland; 3 Barts Health NHS Trust, London, UK; 4 University of Edinburgh, Edinburgh, UK; 5 Northwest London Clinical Commissioning Group, London, UK; 6 Peking University Medical Informatics Center, Beijing, Peoples’ Republic of China; 7 Fred Hutchinson Cancer Research Center, Seattle, Washington, USA

## Abstract

**Background:**

Health information exchange (HIE) is frequently cited as an important objective of health information technology investment because of its potential to improve quality, reduce cost, and increase patient satisfaction. In this paper we examine the status and practices of HIE in six countries, drawn from a range of higher and lower income regions.

**Methods:**

For each of the countries represented – China, England, India, Scotland, Switzerland, and the United States – we describe the state of current practice of HIE with reference to two scenarios: transfer of care and referral. For each country we discuss national objectives, barriers and plans for further advancing clinical information exchange.

**Results:**

The countries vary widely in levels of adoption of EHRs, availability of health information in electronic form suitable for HIE, and in the information technology infrastructure to be used for transmission. Common themes emerged, however, including an expectation that information will be exchanged rather than gathered anew, the need for incentives to promote information exchange, and concerns about data security and patient confidentiality.

**Conclusions:**

Although the ability to transfer health information to where it is most needed is nearly always mentioned as an advantage of HIE adoption, there are wide differences in the degree to which this has been achieved to support the scenarios used in this study. Nevertheless, these differences indicate varying stages of progress along a comparable pathway, with similar barriers being identified in the countries described. In some cases, these have been partially surmounted while elsewhere work is needed. We reflect on contextual factors influencing the status and direction of HIE efforts in different global regions and their implications for progress.

The ability to exchange patient information across clinical contexts is frequently cited as an important objective of health information technology investment because of its potential to achieve goals of improved quality, reduced cost, and increased patient satisfaction [1]. There is hope that as a result of greater information exchange unnecessary duplicated services will be reduced, and the delivery of care will be more coordinated and efficient. These hopes have contributed to extensive efforts globally to implement electronic medical records, to develop the policy and technical infrastructure required for clinical information exchange, to provide incentives for clinicians to use this infrastructure in daily practice, and to establish pilots to assess the use, barriers, and results of information exchange [2]. In some countries, efforts have progressed further than in others and in certain regions HIE efforts extend across national borders.

Unsurprisingly, not all these efforts have been successful, nor have they been extended to cover the care of all citizens of each country [3-5] although many nations have plans to increase the degree and utility of clinical information exchange. In this paper we concentrate on what occurs today in six countries drawn from a range of higher and lower income regions, by analyzing ways in which HIE is enacted with reference to transfer of care between primary care practitioners and referral from primary to secondary/specialist care (either to seek an opinion or transfer care responsibility). The relevant scenarios, shown in **Box 1** and **Figure 1**, represent frequently occurring needs for clinical information transfer in most countries. The selected countries differ in terms of their global economic status, the maturity of their information infrastructures, the structural organization and financing of their health care systems and the extent of government involvement in the health sector.

Box 1 **Scenarios for health information exchange used to guide country comparisons.**

**Transfer of care**. Mr. James Smith has a chronic health condition. He has lived in one city for 20 years and is transferred to another by his employer. He makes an appointment to see the new primary care provider he has chosen. How does the new primary care provider gain access to his prior medical record, so that care proceeds without delay, omission or duplication?
**Referral**. Ms. Mary Jones has been troubled by headaches beginning 6 months ago. Following initial evaluation and diagnostic tests, her primary care provider refers her to a neurologist that they jointly selected. How does the neurologist gather studies needed for the evaluation (records of visits, MRI, laboratory results), or are new studies performed?

**Figure 1:**
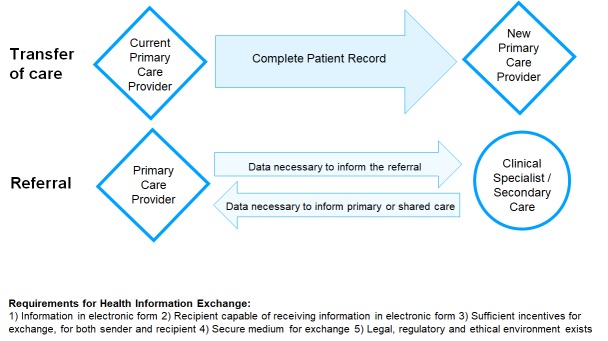
Exemplar health information exchange scenarios.

For each country, we describe current HIE practices and infrastructure, as well as reflecting on how these have evolved in recent years. We also describe some of the barriers to HIE (technical, structural, cultural and political) that have been overcome and those which may explain why the extent of clinical information exchange today falls short of stated national goals. The overarching objective of the paper is to learn lessons from the national experiences of each country, to complement the discussion of national objectives and plans for advancing HIE [6].

Although the term ‘Health Information Exchange’ is associated with multiple concepts [7,8], *for the purposes of this paper, we focus on the administrative, technical and behavioral processes involved in the secure transfer of patient information from one clinical setting to another*. Throughout the paper we use Health Information Exchange and Clinical Information Exchange synonymously, depending on the context.

## METHODS

Each author gathered information from the country of their expertise (and in most cases, current residence) by review of published and unpublished literature, government reports, and consultations with colleagues in the field. Authors were selected for their involvement and connections in health informatics from an academic, policy or professional standpoint, or through a combination of these connections. The scenarios used to scaffold this exercise were initially developed for an international HIE workshop delivered by the authors at the Medinfo 2013 conference in Copenhagen, Denmark. The country status reports have been reviewed and updated periodically since that time by the authors and their contacts, most recently in April 2019 during the development of this manuscript, which also benefits from peer-review. To the best of our knowledge, they provide an up-to-date summary of current practices, mindful of the caveats noted in the discussion.

## RESULTS – CURRENT STATE IN EACH COUNTRY FOR THESE SCENARIOS

### China

#### Description

According to official statistics, by the end of November of 2018, there were a total of 32 476 hospitals (12 072 public and 20 404 private) in mainland China (not including Hong Kong Special Administrative Region, Macao Special Administrative Region and Taiwan Province). These hospitals serve the entire Chinese population with specific designations, in accordance with the hospital capacities and regions they serve. Most of the public and some of private hospitals are assigned one of three possible tiers, with the tertiary grade considered to be the best hospitals. In total, there are about 2498 level III hospitals (major medical centers, provincial hospitals, and teaching hospitals of medical universities), about 8806 level II hospitals (municipal or county hospitals), and about 10 477 level I hospitals (township or community Hospitals). In the past, China had three kinds of basic medical insurance: basic medical insurance for workers, insurance for the new-rural cooperative medical system, and basic medical insurance for urban residents. Since 2016, the State Council has started integrating basic medical insurance for urban residents and insurance for the new-rural cooperative medical system and establishing a unified basic medical insurance system for both urban and rural residents. On May 31 of 2018, China's new State Medical Insurance Administration was officially set up in Beijing. Currently, these insurance schemes cover over 1.3 billion of the Chinese population. To ensure appropriate operation of the large insurance program, the government has established various programs to improve secure sharing of health care information.

#### Infrastructure

As China is undergoing modernization, the Chinese government has recognized the importance of reforming the health care system and has implemented several waves of health care reforms over the past two decades. Amongst all reform strategies, leveraging health care IT has been a key component. Since the 1980s, the development of the health care IT infrastructure has gone through four stages. During stage 1 from 1980 to 2000, many major hospitals in the economically advanced areas built hospital specific systems, to cover registration and billing. In stage 2 from 2000 to 2005, most level III hospitals, with a few smaller hospitals, implemented the hospital information management system that integrated information from hospital administration and management. During stage 3 from 2005 to 2010, most level III hospitals devoted significant resources to the implementation of electronic medical records (EMR) systems, and some began to use Hospital Enterprise Resources Planning (HERP) systems. Health information systems are now used in essentially all of level II and III hospitals. In the meantime, early electronic health records (EHR) systems began to build up in some level I hospitals, public health and primary care institutes. Since 2010 and the onset of stage 4, the Chinese government has pushed forward health care reform proposing that this be based on EMR & EHR technologies and delivering improved care for people in all socioeconomic classes. Unlike previous health care reforms, sizable funding has been allocated to the development and implementation of hospital information systems. Increasingly, developed areas, such as Beijing, Shanghai, Zhejiang Province, Guangdong, Shandong, Fujian, Henan Province, local government has devoted resources to build regional health care information networks and rural and community health information platforms. Many experiencing these developments have achieved preliminary success through sharing health care information locally or regionally. Meanwhile, some large health care organizations through mergers and acquisitions have also developed their organizational health IT infrastructure to improve data sharing.

China has undergone enormous economic development in recent years and has accomplished a great deal in the health IT area. However, many issues remain, presenting challenges and barriers to the further development of health care IT. Some of these barriers are unique to China, due to its differences in health care structures and insurance policies in comparison with other countries. For example, while the development of hospital information systems in level III hospitals has resulted in internal integration of data sources and achievement of the objective of data sharing in real time, sharing data across hospitals is still incomplete. In common with experiences in other countries, however, heterogenous IT structures, architecture, database technologies, database systems, and data standards also remain significant barriers. While both central and local governments have devoted substantial funding to support robust health care information sharing across large regions, few regional HIE systems are fully functional today.

There is a somewhat unique feature of Electronic Health Records (EHR) in China, with somewhat different definitions from commonly adopted definitions. For hospitals of level 2 or 3, electronic medical records system (EMRs) are routinely adopted to manage all pertinent medical information, internal clinical information exchanges, patient care and services. In contrast, the level 1 hospitals (and also those primary health clinics throughout countryside or local communities) have adopted Electronic Health Record systems (EHRs), which are constructed for managing and sharing basic medical and health information among hospitals in the region and, most importantly, for public health information management. While the EMRs and EHRs share some medical-related contents, they have been optimized for fairly different objectives and hence include many components that are fairly different. Currently, most of these two systems are independent of each other, and are not inter-connected. Through recent promotion and organization of the National Health Commission of China (formerly the Ministry of Health), EMRs in level 2 and 3 hospitals have been generally adopted, and are well developed for hospital operation, management, patient-care, and patient services inside the hospitals. Indeed, the development of health information technology is an important criterion for hospital evaluation and for designation of hospital competencies. In contrast, EHR systems in level 1 hospitals or other health clinics have not been developed as well, despite major effort by governmental promotions and supports. There remain substantial gaps and challenges for health data exchanges between regions, even though some within-region exchange systems are reasonably developed. Additionally, even for those well-developed regions, information sharing between EMR and EHR is still lacking. A primary contributing factor for this lack is that EMRs and EHRs are generally developed by different groups of companies in different years. Because of different requirements and purposes for developing EMR and EHR systems, these companies have adopted different information standards, infrastructures, and data formats for their respective objectives. Consequently, these two systems are not easily integrated, and the differences present a major challenge for health information and data sharing across hospital-oriented and public health-oriented systems for a foreseeable future.

#### Incentives

Most hospitals are public and are overloaded in meeting the country’s health care needs. The primary incentives for hospital leaders who are taking on the development of health IT are reputational gains and recognition for being progressive in adopting information technologies and actively engaging in the health care reform.

#### Transfer of care scenario

As noted above, China is large and quite diverse in economic development across regions. Suppose that Mr. James Smith is living in a relatively more developed area where the regional health care IT has been built, for example Shanghai, and that all of his medical records are stored within EHR system of his primary care facility. Now if the EHR system in this primary care facility is consistent within the regional sharing network, Mr. Smith is able to access all of his medical records stored with the old health care provider while receiving care from his new health care providers. When the two EHR systems are not consistent, Mr. Smith can only bring all of pertinent information printed on paper from the older health care provider to the new health care provider. For missing information, Mr. Smith can provide verbal recollection. While verbal recollections of medical history are useful for guiding the physician’s judgement, they are unlikely to be stored in the new EHR system. At this time, it is hard to estimate what percentage of EHR are shared.

#### Referral scenario

Consider the current scenario that Ms. Mary Jones has a headache for more than six months without obvious reason and that the primary care physician could not provide any definitive diagnosis. Ms. Jones is then referred to secondary and tertiary hospitals to see a neurological specialist. Given the current status of health care IT in China, it is likely that she would bring a paper-based medical record and may travel many hours or even days to a larger hospital. After a long wait in the large hospital, she would be registered, and then wait hours for her turn to see the doctor. Typically, senior physicians in larger hospitals are very busy, and it is likely they would spend only a few minutes to ask for medical history and symptoms, read the paper-based medical history, and order laboratory work and an MRI. Newly generated clinical data would then be entered into the hospital’s EMR system, while the primary care provider’s information is often not entered into the system. After collecting all pertinent data, the attending physician then provides the diagnosis, and prescribes medication. From this point forward, all health care information for that patient is now stored in that hospital EMR system.

### England

#### Description

The English National Health Service (NHS) was created in 1948 to provide health care free at the point of delivery. The NHS is funded through general taxation. Funds are distributed by parliamentary vote through regional and local organizations for health care. Recently a key organizational change has brought Clinical Commissioning Groups (CCGs) together into Sustainability and Transformation Partnerships (STPs), which usually combine 5 CCGs covering populations of 1-2 million. This has had consequences for health IT expenditure. In 2003, the UK government created the National Programme for IT (NPfIT). The aim of this nationwide initiative was to fully computerize the NHS over 10 years. While the NPfIT did not achieve all of its goals, it did develop a national framework for connectivity and standards implementation, and these have now been adopted by the STPs who have now taken charge of local health IT development and the implementation of a new health information exchange initiative named the Local Health Care Record Exemplar programme (LHCRE). The underlying concepts behind the LHCRE are patient use and ownership of health data, data exchange across traditional boundaries of health economies and service transformation driven by data and system interoperability using clinical and technology standards. For example, the One London LHCRE is designed to provide HIE and service transformation for the whole of London by extending local HIE services, application of interoperability standards for data and image exchange and includes social care organizations.

During the 1980s and 1990s, primary care EHRs exploited the benefits of automated repeat prescribing, disease registries and scheduling. By 2000, nearly 100% of GP and community practices ran fully digital services and reported electronically to the Department of Health. Point of care data collection using a mix of structured and free text data are now the universal standard. An increasing number of practitioners are introducing mobility solutions using cloud-based systems to support district nursing and home based care [9].

The English NHS has developed a connectivity framework:

- NHS Spine – a set of services, applications and directories. The Spine now supports over one billion monthly transactions.
- Health and Social Care Network (HSCN) is the default standard for internet connectivity in the health social care sector. The HSCN works through modern industry standards including use of cloud computing and provides new levels of:
  - Cybersecurity controls,
  - Service management controls,
  - Access to digital services resident on the private NHS network formerly known as N3.
- The Registration Authority – a system for registering clinical and administrative users of NHS IT systems and issuing Smart Cards with role-based access controls.
- NHS Mail – a secure encrypted mail service

The services, applications and directories included in the NHS Spine are:

1. The personal demographic service (PDS). This is a national database of NHS patient demographic details holding a unique patient identifier (NHS number) as well as patient preferences for data usage.
2. GP2GP. This is a HL-7 standard based record transfer service that enables general practitioners to transfer records from one practice to another.
3. The summary care record (SCR). This is a summary record holding patient demographic details and medication history. The SCR can be viewed by SCR compliant GP systems as well as using a national viewing application with a patient consent model applied at the point of care. The latest version of the summary care record extends viewing of clinical information.
4. Electronic prescribing service (EPS). This is a digital prescribing service for patients and general practitioners and supports the digital transmission of electronic prescriptions to patient nominated pharmacies.
5. E-Referral Service. The service is based on hospital directories of service and at present semi-automate referrals from general practice to hospitals. The current system supports the provision of online advice and guidance from hospital teams back to community care teams.
6. Picture archiving and communication service. The HSCN network can be used for image transfer from one hospital to another.
7. The secondary uses service (SUS). This is a repository for healthcare data in England which enables a range of population health initiatives that include payment, public health planning and policy development.

An increasing number of hospital services have implemented enterprise wide health information systems that fully exploit the potential of the connectivity framework. Where these have been implemented progress is now being made in viewing summary EHR pages and data exchange. The barriers to full implementation continue to be those of provider resistance to change, funding at a time of economic austerity and the failure to fully implement informatics standards.

#### Incentives

The current incentives in the system differ in primary and secondary care. In primary care the main incentives for implementing health IT are cost savings to provider owned practices, government requirements for data reporting [10] and primary care based research collaborations. The Quality and Outcomes Framework (QOF) is a voluntary annual reward and incentive program for all GP practices in England. For secondary care, the main incentive during the period of the national program was that software systems were purchased centrally at no cost to the organization other than implementation costs. Recently, national grants have been established based through a competitive bidding process against specific health service goals [11]. In secondary care, these funds have been directed at a program for establishing Digital Exemplars and through a separate national allocation for the Local Health Care Record Exemplars.

#### Transfer of care scenario

After moving, Mr. Smith can ask to register with any NHS primary care provider as long as that provider has spare capacity. The patient’s identity will be confirmed using the tracing tools in the Personal Demographic Service. A message will be sent to the previous practice requesting an electronic copy of the patient’s EHR. If the previous GP practice is GP2GP enabled, then the practice will confirm whether an electronic extract of the EHR can be made and the transfer process will begin. The majority of GP practices are now able to use GP2GP and the transfer of Mr. Smith’s electronic record will be completed using this transfer mechanism from his previous GP’s practice. Transfer of EHRs is now a contractual requirement for GP practices and all practices published a route map to full GP2GP transfer by March 2015. Older patients who may still have paper records will have their paper records transferred by secure courier. Mr Smith can seek to view his record through the GP Connect program. All the major primary care systems can provide patient access to their data and which will be enabled for Mr Smith manually by physical identity checks in the practice.

#### Referral scenario

Ms. Jones and her general practitioner will discuss the need for referral. The GP can refer to any secondary care provider although in practice this choice is usually limited by contractual agreement to local providers and a limited number of specialist centers. The referral to the neurologist will be made using the national e-Referral Service (e-RS) and which gives the patient some control over time and date slots. This service is, however, only semi-automated and it is not currently possible to book specialist clinic time on line using the e-RS. Imaging and scans can be viewed or transferred from provider to provider using the national Image Exchange Portal (IEP) or using local exchange protocols. The receiving neurologist can provide advice and guidance using the e-RS and also arrange to redirect the referral using the national service. Electronic exchange of clinical notes, treatment plans, prescribing information and visit records is significantly less advanced. In local health economies, it is usual to have systems for exchange of laboratory results between hospital providers. Increasingly, local Health Information Exchange platforms are in place which, support information exchange between primary and secondary care. A framework for action to deal with these gaps in delivery has been published [12].

### India

#### Description

India’s population is over 1.2 billion and still growing at a rapid rate. Healthcare expenditure accounts to 2%-3% of GDP. The private medical sector remains the primary source of health care for 70% of households in urban areas and 63% of households in rural areas. Seventy percent of all health care expenditure is out of pocket. Only 5%-10% of the population has health insurance. HIE is virtually non-existent currently.

#### Infrastructure

EHRs and other health care technology solutions are deployed in largely tertiary center and private facilities that cover about 10%-20% of the urban population. The focus of information technology has been largely on administration rather than clinical care. A few private organizations are capturing and exploiting clinical content in centers of excellence.

The Government of India is actively encouraging Health IT adoption. India follows a federated system with Healthcare being a State prerogative. Most of the states have used the framework and policy guidelines created by Govt of India to create systems unique to their states. Some of the examples of the national initiatives are as below:

- A national initiative- Ayushman Bharat [13],
- A national health portal for all hospitals and healthcare providers- this will form the basis of health information exchange later [14],
- A national identification number system for health facilities in India [15],
- An online patient registration system across all states and hospitals [16].

#### Incentives

India exhibits extremes in access to both basic and advanced health care. HIE infrastructure has potential to strengthen public health care systems through connecting records and services, whilst also supporting the growth and accountability of private insurance-based health care. In addition, HIE addresses the inability of leading edge institutions to contribute to meaningful research in spite of the vast clinical data available to them. Healthcare tourism is also a great source of revenue to the country and its growth is fundamentally dependent on the availability of a standard, secure HIE.

#### Transfer of care scenario

A patient-maintained handheld paper record (if they hold one) will be the most reliable source of medical information when moving from one provider to the other or one city to the other. It will most often have the bills and receipts from hospitals and pharmacies which are often the only means of knowing what medicines the patient is taking. Laboratory results are almost always paper laboratory reports and imaging reports are often preserved as x-ray films and sometimes on digital media. Most often a doctor’s source of past medical history is dependent on the patient’s memory, educational status and ability to collate a longitudinal health record. Detailed medical records or discharge summaries are currently only seen in high end private hospitals or teaching institutions and clinicians largely rely on patient recall and previous clinical notes to assess medical history.

#### Referral scenario

There is no infrastructure or standard in place for exchange of clinical information across different providers and across the different states in India and prospects for a unified approach is unlikely due to fierce market competition among private health care services. The state is beginning to mandate minimum standards for EHRs and Consolidated-Clinical Document Architecture (C-CDA) standards since none exist today. Today, the best way to collate all information and results falls to the patient including tracking all repeated tests performed at different sites. There is also a great deal of variation in the quality and validity of laboratory results and, due to substantial variation in the quality of laboratories, many providers don’t trust reports and results from other sources. Prospects for HIE appear limited today since there is no business case for HIE in the private sector and unhealthy competition is the norm.

### Scotland

#### Description

Scotland is small nation within the United Kingdom (population 5.3M and 64M respectively) with a devolved government responsible for the administration of health and social care. It nevertheless shares many features with England, such as a unified National Health Service sponsored through taxation, offering free services at the point of care, the same professional training and governance, and an overlapping IT system supplier base.

Primary care is the first point of contact for patients and is usually delivered in health centers by teams of General Practitioners (GPs), nurses and support staff. All citizens are eligible to register with a general practice and the vast majority are enrolled. Access to planned secondary (hospital or specialty) care is via referral from primary care in most cases.

#### Infrastructure

Scotland made early progress with health IT, particularly in primary care, with the implementation of computerization and clinical coding having taken place over the last two decades. Close to 100% of general practices are now paperless, with in-practice networked information systems including digital patient records, electronic prescribing, decision support, clinical communications and administrative tools. Progress in secondary care has been slower, hindered by regional and local variations in systems and data practices.

At the heart of the existing infrastructure are repositories for medical images (PACS), laboratory results and scanned documents (SCI-Store), 3 regional portals enabling clinicians to view their patients’ health and care records, and a national system for exchanging electronic clinical information such as referral letters and discharge documents between primary and secondary care (SCI-Gateway). Successful Health Information Exchange and record linkage across different parts of the service depend on the use of a unique patient identifier known as the Community Health Index (CHI) number, which is associated with a central population register. A key tool for exchanging patient information amongst various parties involved in the provision of health care services is the Emergency Care Summary record (ECS), containing core information about demographics, prescribed medications and allergies/ADRs. This is extracted routinely from all general practice record systems and can be accessed by out-of-hours and emergency services, as well as community pharmacies and some scheduled care services such as outpatients. Until recently, patients would be asked for their explicit consent to view the ECS at the point of care, but this requirement has been displaced by recent legislation and now only applies for access which is not part of the patient’s health care. In certain patients at high risk, or with complex needs, the ECS is supplemented by a Key Information Summary, with explicit consent required for the addition of details such as conditions and preferences (eg, for end-of-life care).

The national eHealth strategy of 2011-17 envisaged migration of regional portals to a national acute Patient Management System (PMS), to drive convergence and standardization of IT systems and to support clinical information exchange within and between hospitals and eventually between secondary and primary care. Nationwide implementation of the PMS (TrakCare) has proven challenging however, with organizations choosing to approach this in somewhat different ways. Delivery of a new cloud-based National Digital Platform (NDS) is a key objective within the latest eHealth strategy, released in 2018. Based around a central Clinical Data Repository, this aims to provide a single patient-centered health and care record of core data, available to authorized professionals and citizens themselves, supported by a national citizen identity management system and NHS staff identity system. The NDS will use the OpenEHR international standard architecture to support interoperability and provide APIs to enable health providers and patients to access various digital services. Current systems suppliers are seeking to adopt HL7 FHIR messaging standards to interoperate with this platform.

#### Incentives

In primary care the purchase of computers and software has long been supported by central government, up to 2012 through the provision of a national GP information system (GPASS) and subsequently through the direct supply of a choice of two government-approved systems used across Scotland (EMIS PCS and Vision), with a further supplier to be added in 2020 (Microtest). The appropriate use of IT was incentivized by the UK-wide scheme known as the Quality and Outcomes Framework, introduced in 2004, which rewards GPs for documenting Read-coded health care quality indicators using their electronic information systems. In secondary care there are no such payments to clinicians and use of IT systems is driven by the clinical need for rapid access to laboratory results, images and referral letters. Key secondary care data, PACS images and laboratory results are now viewable in the regional portals, with a plan to make these interoperable to support nation-wide access, as a precursor to fully-digital HIE enabled by the National Digital Platform. The current hybrid system of digital, analogue and semi-digital HIE is reflected in the scenarios below.

#### Transfer of care scenario

Mr Smith visits the new GP practice and completes a paper registration form with his name, address, date of birth, the name/address of his previous GP and his Community Health Index number (CHI), if known. The practice enters the details onto its clinical information system, then electronically messages the data to Practitioner Services Division Scotland (PSD) which matches his identity on the CHI database, updates that database and contacts the previous GP practice to request his records. The records are mostly stored as document images and electronically transferred to the new GP, via PSD, using a document transfer service (Docman Transfer). In most cases data from these documents must then be re-entered manually into the new GP system, although a new automated process (GP2GP) is currently being rolled out to enable practices to transfer electronic and scanned information.

#### Referral scenario

Referral from Primary to Secondary care is enabled through use of the CHI identifier. Ms. Jones’ GP securely accesses the SCI-Gateway system via their desktop computer and completes a structured referral template by selecting the service requested, adding details of her clinical presentation, updating the pre-populated medical history and attaching electronic copies of any relevant documents. This is sent via SCI-Gateway to the receiving physician or unit for triage. Using the Regional Portal, PACS and SCI-Store systems the receiving physician is able to access the results of previous laboratory tests or scans. SCI-Gateway can also be used to contact the GP for additional documentation or tests. Ms. Jones is contacted directly by the appropriate clinical service, with a provisional appointment date and a telephone number or email address to use if she needs to reschedule.

### Switzerland

#### Description

Switzerland has a population of 8.45 million. In 2010, life expectancy was 80.2 years for men and 84.6 years for women; there were 4.9 hospital beds per 1000 inhabitants and health care represented 10.9% of the Swiss GDP. In 2008, 13.8% of the employment in Switzerland was related to the health sector [17]. The Swiss health care system has two major pillars: Introduced in 1995, universal coverage is based on a mandatory health plan provided by private companies, and private additional plans providing a wide variety of benefits. Its costs and levels of reimbursement are fixed by the government and insures access to core cares to all citizens with free choice of physicians and care facilities nationwide.

The political organization of Switzerland leaves a very large autonomy to Cantons. There are 26 Cantons in Switzerland, each of them having a ministry of Health and a law for health.

Since 2017, there is a Swiss federal law enforcing a shared patient record. The law places citizen at the center of the process. Citizen can decide freely to have their patient record shared. If so, citizen can decide which document can be accessed, and can grant access to care providers. Inpatient care facilities must be connected to the system by 2022 or might nor more be subject for any reimbursement claims from the universal coverage health insurance system.

#### Infrastructure

The whole process is coordinated by a national coordination committee, under the umbrella of the federal and the cantonal governments as well as major stakeholders. Several working groups work under this committee, including education, evaluation, standards, and architecture. The working group in charge of standards and architecture is proposing technical and functional specifications to help building a distributed and interoperable system based on IHE standards (Integrating the Healthcare Enterprise) for linking across communities. It is also identifying semantic standards, such as LOINC (**Logical Observation Identifiers Names and Codes)** or SNOMED (Systemized Nomenclature of Medicine), that are used and led Switzerland to become a SNOMED International national member. The infrastructure incorporates a set of central services, such as unique patient identifier, index of communities, metadata server, and index of care providers that support ubiquitous interoperability. HIE communities have to comply with standards based on distributed repositories of documents, distributed documents, and patient indexes. A certification process insures compliance with standards as a condition for inclusion in the network. All technical and semantical standards required are available on the eHealth Suisse website [18].

#### Incentives

The incentives are based on several pillars. A) There is a financial support to help inpatients care facilities to become interoperable with the shared record; B) As mentioned above, inpatient acute care by 2020 and long-term inpatient care by 2022 have to be connected with possible penalties on reimbursement by the universal coverage insurance. C) There are many Cantons, such as Canton of Geneva, investing in the shared patient record.

Efforts to improve compliance are under way at several levels. The specifications published by the coordination group are widely used and implemented by vendors. The deployment in several states is ongoing with a growing adoption. While number of users is still very low, it is still positive taken into account that the deployment started in 2013 in Geneva as a pilot Canton. As of Spring 2019, there were 50 000 patients connected, which is a success being known that the deployment was focused on complex cases (The Canton of Geneva as about 800’000 inhabitants).

#### Transfer of care scenario

First, Mr. James has to agree to participate to the Swiss eHealth program. A visiting nurse will discuss and explain the benefits and risks of the shared patient record. If Mr. James agrees to participate, the nurse will help completing forms, registration and consent. Then Mr. James will receive his credentials, such as a SwissID, which allows a strong electronic authentication and all participating care providers will have to send their documents to one of the Swiss eHealth distributed platforms. Mr. James can define who has which role, such as treating physician, and especially the trusted physician. When a trusted physician accesses the record of Mr. James, he will have access to all documents published by any other provider in the appropriate access level. There are several roles possible, and 4 confidentiality levels, from administrative, very open, to secret, eg, available only to the patient.

A primary care shared patient record is starting to be established in Switzerland based on the Integrating Healthcare Enterprise cross-community access rules. Each provider can be part of at least one community. Communities are certified by the federal government. Patients can access all their documents, whichever community holds them, and from any community portal.

#### Referral scenario

It is considered that Ms. Jones and all her care providers participate to the Swiss eHealth program. When Ms. Jones selects the neurologists with her GP, she will be able to grant the neurologist the appropriate access level. At that moment, the neurologist will have access to all previously published documents related to the headache problem. Thus, the neurologist will be able to schedule any required investigation prior to Ms. Jones’ first visit. When the encounter takes place, the neurologist and Ms. Jones can access all required documents, including personal notes, documents, data from quantified-self, etc. that Ms. Jones could have uploaded before.

However, if Ms. Jones does not participate to the Swiss eHealth program, which is still the case for most Swiss citizens, or if the care providers does not publish the documents to the Swiss eHealth platform, which is also still the case for most care providers in Switzerland, then only traditional clinical transmission will occur.

The strategy, the law, the standards and architecture to move toward a universal digitalized shared patient record in Switzerland are well defined. Technical solutions are available, as well as implementations. However, roll-out is at its beginning, with some Cantons, such as Canton of Geneva, Basel, being early adopters.

### United States

#### Description

The 328 million American citizens receive health care through a wide variety of care settings and coverage mechanisms, and many (29 million in 2017) are uninsured [19]. The Patient Protection and Affordable Care Act of 2010 has changed health insurance coverage patterns to varying degrees, and its implementation has been politically and technically difficult.

#### Infrastructure

Since the American Recovery and Reinvestment Act was passed in 2009, there has been rapid growth in adoption of electronic medical records in US clinic and hospital care settings where Americans receive care as a result of Meaningful Use incentives programs [20]. For example, adoption of EHR systems in hospitals has more than doubled since 2009. (Adoption is lower in hospitals ineligible for federal Meaningful Use incentives.[21]) Stage 1 and Stage 2 Meaningful Use requirements [22] led not only to rising EHR use, but in creation of transfer of care documents and in providing patients with electronic summaries of care. In addition, some EHR vendors have created capabilities to exchange information with other sites that also use their products, and to a lesser extent, vendors have supported exchange of clinical information between EHRs from different EHR vendors. The US Office of the National Coordinator for Health Information Technology (ONC) has led development of national standards to exchange information including the Nationwide Health Information Network [23] and through electronic mail-like systems (Direct [24]), regional health information exchanges, and in other ways. Ten years after the American Recovery and Reinvestment Act, interoperability is a major focus of ONC [25]. Historically ONC has described 3 types of exchange: directed exchange – ability to send and receive secure information electronically between care providers to support coordinated care; query-based exchange – ability for providers to find and/or request information on a patient from other providers, often used for unplanned care; and consumer mediated exchange – ability for patients to aggregate and control the use of their health information among providers [26]. As a result of all these changes, and others, current levels of HIE are changing rapidly [27,28]. Currently most of the clinical data exchange in the ambulatory setting occurs between providers within the same organization [29]. Sharing of laboratory results and imaging reports is most common, but exchange of notes and patient summaries is growing.

#### Incentives

Attesting to Meaningful Use requirements carried substantial financial benefit – up to USD 44 000 per eligible provider and far more for eligible hospitals. Growth of alternatives to fee-for-service payment such as Accountable Care Organizations, payment for quality, capitated care and other mechanisms where financial risk is shared provide incentives to organizations and providers to avoid clinically unnecessary duplication of studies and services. Much outpatient care is paid as fee-for-service and inpatient care is paid largely based on Diagnosis Related Groups, which provides a financial incentive to reduce clinically unnecessary duplication of services. Some surgical procedures, such as joint replacement, are reimbursed using bundled payments, which also provide an incentive to reduce clinically unnecessary services [30]. Use of bundled payment policies is growing as part of the Center for Medicare & Medicaid Innovation Center [31].

#### Transfer of care scenario

As of this writing, when a patient such as Mr. Smith transfers care from a primary care in on location to another, the transfer of health information will be different if he moves within an integrated care delivery system such as the Department of Veterans Affairs or Kaiser Permanente. Transfer of information will also be different if his new primary care practice uses the same EHR vendor as his previous primary care site. Some EHR vendors permit electronic transfer of notes and care summary documents, though reconciliation of the new and old record requires manual review. However, exchange of his record between locations of care that are not within the same delivery system or not between sites sharing use of a vendor that offers electronic transfer between customer sites, then record transfer will most likely occur through fax or as print copies sent via US mail.

#### Referral scenario

When referral for consultation occurs *within* a health care organization using the same EHR such as mentioned in Transfer of care scenario, electronic transmission of the referral is common. The consultant would have access to records of visits, MRI and laboratory results and other clinical data. However, if the consultant is *outside* the organization or uses a different EHR (more than one EHR is sometimes used within a health care organization [32]), then estimates of electronic HIE is about 50% [33] though this percentage is rising. Published data from 2011 showed that only 31% of physicians were electronically exchanging clinical summaries with other providers [34], (though that percentage is also rising [29]) and that further exchange was happened by lack of interoperability of most EHRs [35]. As in Transfer of care scenario, if the consultant and primary care physician both use an EHR from a vendor supporting record transfer between customers, then most of the patient’s record could be transferred electronically.

## DISCUSSION

**Figure 2:**
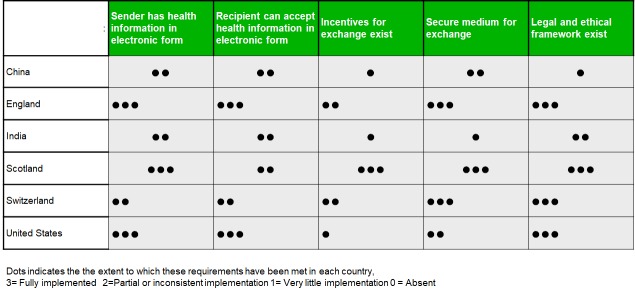
Requirements for clinical information exchange and their state in six countries.

The World Health Organization (WHO) eHealth Resolution includes the aim to *“to foster exchange of data and information for promotion of health, health systems, and training of health-care workers* [36].” As these comparative scenarios illustrate, effective and efficient electronic Health Information Exchange is an important health system strengthening goal for most countries, although they vary considerably in the extent to which this has been achieved (**Figure 2**). Here we reflect on some of the similarities and differences across the six countries represented in this manuscript, considering not only the technical details but also the wider policy and technological context of health information exchange, drawing on the experience of the authors.

### Infrastructural capability and data quality

The technical infrastructure required for HIE includes electronic medical record systems or other systems to gather clinical information, both at the point of capture and at the recipient organizations, alongside secure networks over which the information is transferred. Countries vary widely in the availability and scaling of HIE infrastructure, and the maturity of HIE processes, even within countries whose health systems are already highly digitized.

Obtaining the most clinical value from health information exchange depends on records being present in electronic form. While some countries have or are near to implementing fully digitized, interoperable patient data, in others being able to use the available HIE technology for transferring clinical information depends on forms being printed, scanned and then transferred, which depletes their usefulness for secondary analytics or potential for integration with other information systems. According to the model outlined by Walker and colleagues,^1^ transmission of scanned documents corresponds to the most basic level of information exchange. It is therefore somewhat surprising that even some advanced health informatics environments have been slow to adopt these approaches. Efforts to collect, scan and route this information compete with other obligations for clinical staff, thus while HIE may be possible, it may not always occur. In other parts of the world the onus is still upon the patient to maintain a timely paper-based record and manually transport that to different health care providers, even where computer systems are available, and the exchangeability of electronic data may be very different within institutions than across them. Thus, in some countries, specialist private health organizations represent digital islands of excellence within an otherwise information-poor environment. Many countries are beginning to place a stronger emphasis on the patient as a central player in the HIE ecosystem, with several having implemented, or being in the process of implementing Personal Health Records that allow greater accessibility, choice and opportunities to contribute supplementary data from health devices or apps.

### Incentives

Financial and other incentives for information exchange also act as drivers or barriers to HIE adoption. Whilst financial incentives are being widely used in some higher income countries to encourage electronic records, data coding and HIE, these are not universally applied within health systems, raising equity issues. However international experience suggests that HIE adoption will also be motivated by other types of incentive, such as clinicians’ desire to provide better care, managers’ desire to improve efficiency, or patients’ expectations or aspirations that their information will be shared under appropriate circumstances, rather than having to be repeated.

### Security and privacy

Although not explicitly addressed by the clinical scenarios used in this exercise, concerns have been expressed about information security and privacy in all six of the countries considered [37]. Although there is a widespread desire, or even expectation, that clinicians will have relevant patient information available at the point of care, the mechanisms for enabling this (electronic medical records, transmission across networks) create inevitable security risks, whilst a lack of clarity about who may be permitted to view confidential information can have negative effects on patient trust. These concerns are raised by the increasingly regular reporting of medical data theft and privacy breaches [38,39]. Citizens of certain countries may also be affected, in different ways, by the re-use of their health information for the purposes of informing business efficiencies, medical research, government analytics, or private health care insurance, depending on the political, economic, legal and cultural context of health care. As major HIE systems begin to scale worldwide and as pressure grows to share data across national boundaries for medical research and business analytics, these issues around trust and security are likely become ever more critical for policy makers. The move towards Personal Health Records systems, noted in several of our country profiles, has potential to improve this trust by enabling greater transparency and choice about information-sharing.

### Health system structure & competition

HIE is significantly harder to achieve in disaggregated health systems characterized by high levels of competition between providers. Indeed, this was one reason for the US government’s various incentive programs for HIE and EMRs [20]. As described in our summary of the Indian HIE context, organizational competition has almost completely shut down conversations about the possibility of national HIE systems or standards. In China, by comparison, top-down government mandates for greater health information management are contrasting markedly with the reality of segmented health care systems. Even in apparently unified national health systems, political and professional preferences can shape the context of HIE, for example in the UK, policies have fluctuated between favoring centralized national systems and multiple vendor systems, with recent moves towards cloud-based infrastructure offering something in between. It is also important to recognize that HIE is just one approach for advancing patient-centered care and information fragmentation, which also includes other strategies such as Health Record Banking [40] and the Patient Centered Medical Home in the US [41]. Major changes in health system organization are also leading to new models of HIE. For example, the Government of India recently committed to delivering the world’s largest public health insurance scheme. In the absence of an existing digital health records infrastructure, innovative approaches to HIE are being considered, such as blockchain-mediated personal health records linked to the national ID scheme [42]. Similar innovations are also being proposed elsewhere for supporting HIE in decentralized health systems [43].

### Limitations

While this exercise encompasses a range of higher and lower income regions it does not claim to be fully representative of the international HIE environment and other countries’ experience may be different than those of the six countries we describe. Within both high- and lower-income countries, there may also be considerable variation between regions or organizations in the degree to which HIE is occurring; particularly in mixed public/private health care economies, such as China. This heterogeneity would benefit from a further exploration. Although we made an effort to select authors with relevant academic or clinical knowledge about HIE in the countries they describe, all of whom consulted with relevant colleagues, others within each country may have different perspectives. Likewise, what we report here is only a snapshot of extant practices and would benefit from extension to other areas of health care practice alongside ongoing analysis in this rapidly changing area. Using scenarios to scaffold our comparisons helped to draw out some of the wider political and sociotechnical contexts of HIE, in addition to clinical use cases, but it was not explicitly designed to do so, and many important contextual issues are under-represented in these largely technical descriptions. It is possible that information that is publicly accessible and that provided to the authors may not accurately characterize the state of information exchange in each country. Despite these limitations we found these comparisons to be useful in better understanding the state of HIE today in countries other than our own and the exercise provided a catalyst for broader discussions, which we have drawn upon above alongside observations reported in the literature. We welcome correspondence from the wider pool of global HIE experts, able to provide comparable scenario-oriented reports from regions not represented here, particularly Africa and Asia-Pacific.

### Policy implications

The experience of these six countries (5 if considering the UK as a whole) provides some insight for policy makers interested in increasing health information exchange. It is noteworthy that in countries that have successfully achieved HIE, or are on course to do so, the impetus came from government and the change was galvanized with economic incentives to health care providers. This includes both the changes needed to achieve digitized patient records, which are a necessary prerequisite for HIE, and those required to create the infrastructure, policies and processes necessary for information to move between providers. Leadership from government is important, as HIE can challenge traditional organizational boundaries, professional practices and, in some cases, business models.

These approaches must be balanced against the financial demands of delivering health care and the risks of failure that characterize large and complex health IT projects. However, experience indicates that with clear government commitment and provider buy-in, EHR vendors and health care systems will adapt their processes in response, particularly when adhering to common interoperability requirements becomes essential for their viability.

It is worth remarking that, even with political will, finances, technologies and standards for enabling HIE, the complex landscape of health care systems will continue to present barriers to HIE. This may be equally problematic in well-established health systems dealing with legacy software and entrenched data practices, as in countries with little existing infrastructure, who also have opportunities to leapfrog to new technologies, such as mentioned above.

While this did not emerge as a strong theme in the scenarios used to scaffold our HIE descriptions, one of the major incentives driving HIE initiatives is to enable better data for research, management and policy. This aims to drive data-driven ‘learning health systems’, clinical and business insights and medical innovation, providing strategic alignments with other policy priorities, such as public-sector finance and economic growth. For example, using health data to train artificial intelligence is high on the policy agenda of many European countries [44].

## CONCLUSIONS

The implementation of HIE is largely driven by a desire to optimize health care quality and outcomes through having relevant, timely and accurate patient information available at the point of care, although facilitating secondary uses of data for research and analytics represents a parallel objective. Today there are wide differences in the degree to which this has been achieved to support the clinical scenarios considered in this study. Generally speaking, these differences represent varying progress along a similar trajectory. Many similar barriers have also been encountered which, in some cases, have been partially surmounted and in others still loom ahead. Better understanding of the steps along this pathway - adoption of electronic records, establishment of health information infrastructure, creating appropriate incentives and engaging society - may enable these goals to be achieved more rapidly. However, health systems around the world differ on important structural, cultural, economic and political dimensions, which are likely to influence the future development, use and governance of HIE. For this reason, aligning international HIE strategies around a common framework, such as the UN Sustainability Goals, would be worthwhile although political will and the use of appropriate incentives are key to achieving these aims. In addition, further research and ongoing assessment is required to strengthen the existing evidence-base on the impacts and value of HIE for different stakeholders [29].
